# Habitat predictors of genetic diversity for two sympatric wetland‐breeding amphibian species

**DOI:** 10.1002/ece3.3203

**Published:** 2017-07-03

**Authors:** Anna M. McKee, John C. Maerz, Lora L. Smith, Travis C. Glenn

**Affiliations:** ^1^ Warnell School of Forestry and Natural Resources University of Georgia Athens GA USA; ^2^ Joseph W. Jones Ecological Research Center Newton GA USA; ^3^ Department of Environmental Health Science University of Georgia Athens GA USA; ^4^ Present address: U.S. Geological Survey South Atlantic Water Science Center Norcross GA USA

**Keywords:** allelic richness, *Eurycea*, heterozygosity, isolated wetlands, landscape genetics, *Lithobates (Rana)*, longleaf pine, microsatellite

## Abstract

Population genetic diversity is widely accepted as important to the conservation and management of wildlife. However, habitat features may differentially affect evolutionary processes that facilitate population genetic diversity among sympatric species. We measured genetic diversity for two pond‐breeding amphibian species (Dwarf salamanders, *Eurycea quadridigitata*; and Southern Leopard frogs, *Lithobates sphenocephalus*) to understand how habitat characteristics and spatial scale affect genetic diversity across a landscape. Samples were collected from wetlands on a longleaf pine reserve in Georgia. We genotyped microsatellite loci for both species to assess population structures and determine which habitat features were most closely associated with observed heterozygosity and rarefied allelic richness. Both species exhibited significant population genetic structure; however, structure in Southern Leopard frogs was driven primarily by one outlier site. Dwarf salamander allelic richness was greater at sites with less surrounding road area within 0.5 km and more wetland area within 1.0 and 2.5 km, and heterozygosity was greater at sites with more wetland area within 0.5 km. In contrast, neither measure of Southern Leopard frog genetic diversity was associated with any habitat features at any scale we evaluated. Genetic diversity in the Dwarf salamander was strongly associated with land cover variables up to 2.5 km away from breeding wetlands, and/or results suggest that minimizing roads in wetland buffers may be beneficial to the maintenance of population genetic diversity. This study suggests that patterns of genetic differentiation and genetic diversity have associations with different habitat features across different spatial scales for two syntopic pond‐breeding amphibian species.

## 1 INTRODUCTION

The maintenance of population genetic diversity is widely accepted as important to the conservation and management of wildlife. Genetic diversity is important for enabling populations to face environmental challenges. Variable populations have a broader repertoire of potential responses to ambient changes, and subpopulations acting as refuges may make populations more resilient to local extinctions. Additionally, variable populations have a reduced risk of inbreeding depression caused by an increased frequency of deleterious alleles in the population. Effective maintenance of genetically diverse populations requires understanding the evolutionary processes responsible for determining gains or losses of genetic diversity. Over an ecological time frame, genetic diversity in populations is gained by gene flow from other populations and lost through genetic drift (Cleary, Fauvelot, Genner, Menken, & Mooers, 2006; Vellend, 2005). Although natural selection is also a mechanism of evolution, the effect of selection may be difficult to predict (Vellend & Geber, 2005). Therefore, management efforts to maintain or increase genetic diversity in populations should focus on maximizing gene flow by maximizing potential for dispersal and minimizing genetic drift by maximizing effective population sizes.

Gene flow is affected by a number of factors, including species’ life history traits, vagility, and habitat restrictions (Manel, Schwartz, Luikart, & Taberlet, 2003; Slatkin, 1987; Storfer et al., 2006). For a given species, some habitats may be more suitable for dispersal thereby facilitating gene flow, whereas other habitats may be less suitable and restrict gene flow (Cushman, 2006; Manel et al., 2003). Few studies have compared landscape genetics of multiple species within the same landscape (Waits, Cushman, & Spear, 2016). Investigating associations between habitat features and genetic diversity for multiple species within a meta‐community may provide insight on the variability in these associations between sympatric species. This study addressed the following objectives for two sympatric, pond‐breeding amphibian species that occur in the southeastern United States: assess population structures, identify the habitat features most strongly associated with genetic diversity, and examine the spatial scale at which habitat features are most strongly associated with genetic diversity.

Amphibian species that breed in isolated wetlands are appropriate focal organisms for studying patterns of genetic diversity because many occur as metapopulations due to their relatively limited vagility (Blaustein, Wake, & Sousa, 1994; Gibbs, 1998), high philopatry, and fidelity to breeding sites (see Smith & Green, 2005 for review), and because wetlands are relatively small discrete entities embedded in large matrices of terrestrial habitats (Marsh & Trenham, 2001; Smith & Green, 2005).

Many studies have investigated the associations between habitat features and pond‐breeding amphibians. Local wetland characteristics, such as hydroperiod (Pechmann, Scott, Whitfield Gibbons, & Semlitsch, 1989; Skelly, 1996; Snodgrass, Komoroski, Bryan, & Burger, 2000), predator guilds (Gunzburger & Travis, 2004; Murphy, Dezzani, Pilliod, & Storfer, 2010; Piha, Luoto, Piha, & Merilä, 2007), plant communities (Cohen, Maerz, & Blossey, 2012; Maerz, Cohen, & Blossey, 2010), and abiotic conditions (Cohen et al., 2012), are all linked to amphibian performance within wetlands. Landscape features surrounding wetlands such as forests, agriculture, and roads are also related to amphibian population persistence and community richness (Eigenbrod, Hecnar, & Fahrig, 2008; Gagné & Fahrig, 2007; Guerry & Hunter, 2002; Houlahan & Findlay, 2003; Piha et al., 2007; Pope, Fahrig, & Merriam, 2000; Scribner, Arntzen, Cruddace, Oldham, & Burke, 2001; Simon, Snodgrass, Casey, & Sparling, 2009; Skelly, Werner, & Cortwright, 1999). However, less is known about how local habitat and landscape features affect genetic diversity within amphibian populations. Studies suggest habitat features have similar effects on amphibian population genetic diversity and species diversity (Emaresi, Pellet, Dubey, Hirzel, & Fumagalli, 2011; Reh & Seitz, 1990; Scribner et al., 2001); however, most of these studies have focused on a single species without the opportunity to evaluate how landscape features affect genetic diversity among different species within the same landscape (but see Goldberg & Waits, 2010a; Richardson, 2012; Sotiropoulos et al., 2013).

**Figure 1:**
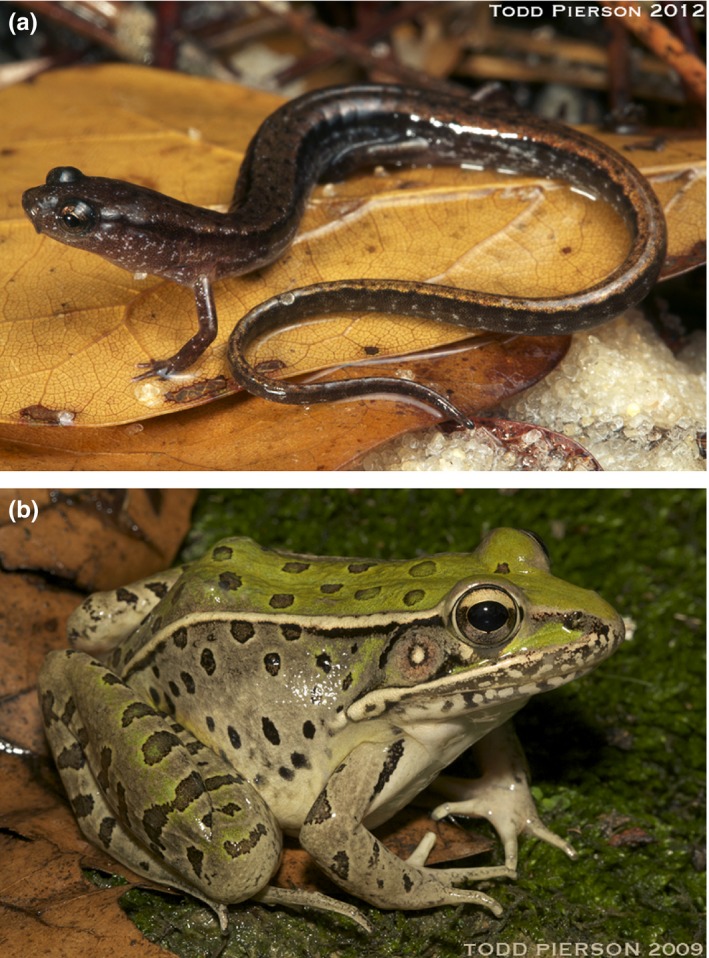
Study focal species. (a) Adult Dwarf salamander, *Eurycea quadridigitata*. Photo credit: Todd Pierson, 2012. (b) Adult Southern Leopard frog, *Lithobates sphenocephalus*. Photo credit: Todd Pierson, 2009

Dwarf salamanders (*Eurycea quadridigitata*; Figure 1a) and Southern Leopard frogs (*Lithobates sphenocephalus*, formerly *Rana sphenocephala*; Figure 1b) were selected as model organisms for this study. Both species are widespread and abundant in the southeastern United States (Cash, 2008; Means, 2008), and as adults, both species are strongly associated with wetlands and wetland edges during breeding and nonbreeding seasons. Although they both utilize aquatic habitats for mating and larval development and semi‐terrestrial habitats as adults, they differ in vagility and microhabitat requirements. Dwarf salamanders have limited dispersal ability (Pechmann, Estes, Scott, & Gibbons, 2001) and more specialized microhabitat requirements (Mount, 1975; Petranka, 1998). Adult Dwarf salamanders are only 22–26 mm snout–vent length (SVL; Means, 2008). Dwarf salamanders are lungless and breath by exchanging gasses through highly permeable and moist skin, making them extremely vulnerable to dehydration and restricting their activity to brief periods proximate to rain events (Feder, 1983). The larval stage of Dwarf salamanders is 5–6.5 months (Semlitsch, 1980). In Baker County, Georgia, Dwarf salamanders have been found in cypress‐gum swamps and grass‐sedge marsh wetlands, which have longer hydroperiods than cypress‐savanna wetlands where Dwarf salamanders were not detected (Liner, 2006). In contrast, Southern Leopard frogs have a greater dispersal ability (Smith & Green, 2005) and breed in a wide variety of wetland types (Liner, 2006). The larval stage of Southern Leopard frogs is around 3 months (Ashton & Ashton, 1988), approximately half that of Dwarf salamanders. Southern Leopard frogs are medium‐sized anurans (adults are generally 50–130 mm SVL), and juveniles and adults have lungs and powerful legs. The lower surface area to volume ratio of a larger‐bodied species reduces water loss, enabling them to be active for longer periods and in drier conditions compared to Dwarf salamanders (Lindstedt & Boyce, 1985). Southern Leopard frog tadpoles are less susceptible to predation by native fish species than other wetland amphibians (Gregoire & Gunzburger, 2008; but see Werschkul & Christensen, 1977), enabling them to breed in sites with predatory fish (Babbitt, Baber, & Brandt, 2006; Baber, 2001). Because of differences in dispersal ability and habitat tolerances, Dwarf salamanders were expected to have greater population structure than Southern Leopard frogs (i.e*.,* more discrete populations as a result of lower dispersal rates among wetlands), and Dwarf salamander genetic diversity was expected to be more closely associated with habitat features at smaller spatial scales relative to Southern Leopard frogs (Antonovics, 1976).

## 2 MATERIALS AND METHODS

### 2.1 Study area

**Figure 2:**
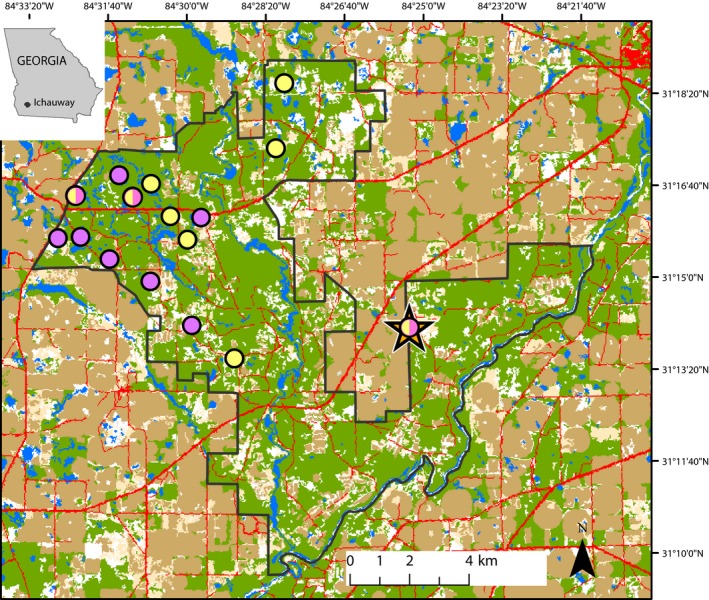
Location and landcover (National Land Cover Data, 30‐m pixels; Homer et al., 2004) at the Joseph W. Jones Ecological Research Center at Ichauway in Baker, Co., Georgia and the surrounding area, and spatial distribution of sample locations for Dwarf salamanders (*Eurycea quadridigitata*; yellow circles), Southern Leopard frogs (*Lithobates sphenocephalus*; purple circles), and both species (yellow and purple circles). The orange star indicates wetland PSK. The black outline is the Ichauway property boundary; blue areas are wetland habitat; green areas are forested upland habitat; beige areas are agricultural land, and red areas are developed land (primarily roads)

Study sites (amphibian breeding wetlands) were located at the Jones Ecological Research Center at Ichauway (16R 740322‐m E and 3456877‐m N; Figure 2) in Baker County, Georgia. Ichauway is an 11,800‐ha longleaf pine (*Pinus palustris*) reserve containing numerous isolated wetlands that vary in size (0.2–76.4 ha), hydroperiod (number days per year the wetland is at least 25% full; 11–225 days), and vegetation type (grass‐sedge marshes, cypress savannas, and cypress‐gum swamps). The properties surrounding Ichauway are composed almost entirely of center‐pivot agricultural fields (Figure 2).

### 2.2 Field sampling

Sample collection began in 2008; however, drought conditions at Ichauway necessitated a second year of sampling. Between April and July in 2008 and 2009, 16 wetlands at Ichauway were sampled using a combination of dipnet sweeps, funnel traps, and cover object searches (described below). Attempts were made to collect at least 30 larval Dwarf salamanders from nine wetlands and at least 30 larval Southern Leopard frogs from 10 wetlands (Table 1, Table S1) across the 2 years. When necessary, adult Dwarf salamanders were collected to supplement the sample sizes.

**Table 1:** Summary of population parameters in nine populations of Dwarf salamanders (*Eurycea quadridigitata*; Eu) and 10 populations of Southern Leopard frogs (*Lithobates sphenocephalus*, Li) at Ichauway in southwest Georgia, USA. Genetic diversity parameter estimates from 12 microsatellite loci in the Dwarf salamanders and 11 microsatellite loci in the Southern Leopard frog. *N* is the sample size after removing full siblings, *r*_g_ is the mean number of alleles rarefied to 24 individuals (smallest sample size, Dwarf salamander) and 13 individuals (smallest sample size, Southern Leopard frog) ± the interlocus standard error, *H*_e_ is the expected heterozygosity (calculated as Nei's unbiased gene diversity; Green, Hooten, Grant, & Bailey, 2013) ± interlocus standard error, and *H*_o_ is the observed heterozygosity ± interlocus standard error. *F* is the fixation index (inbreeding coefficient) with values ranging from −1 to 1. Substantial negative values indicate an excess of heterozygotes, whereas substantial positive values suggest inbreeding or undetected null alleles. *p*‐Value is from Hardy–Weinberg equilibrium (HWE) exact tests in genepop v4.2

Population	*N*	*r*_g_	*H*_e_	*H*_o_	*F*	*p*‐Value
Eu0^a^	30	6.68 (±0.87)	0.65 (±0.09)	0.58 (±0.08)	0.08 (±0.05)	<.001
Eu1^a^	31	6.32 (±0.61)	0.63 (±0.08)	0.57 (±0.08)	0.11 (±0.06)	<.001
Eu3^a^	30	6.89 (±0.71)	0.67 (±0.08)	0.59 (±0.09)	0.13 (±0.06)	.0003
Eu4	30	7.15 (±0.83)	0.69 (±0.06)	0.64 (±0.07)	0.07 (±0.05)	.023
Eu11	29	6.11 (±0.63)	0.61 (±0.08)	0.55 (±0.08)	0.05 (±0.06)	.099
Eu52^a^	29	6.81 (±0.69)	0.67 (±0.07)	0.58 (±0.06)	0.11 (±0.05)	<.001
Eu58^a^	27	5.21 (±0.45)	0.66 (±0.06)	0.57 (±0.07)	0.10 (±0.06)	.0004
Eu68^a^	31	6.85 (±0.64)	0.69 (±0.06)	0.60 (±0.05)	0.11 (±0.05)	.0018
EuSK	28	4.43 (±0.38)^b^	0.62 (±0.05)	0.59 (±0.07)	0.08 (±0.08)	.0287
Li1^a^	31	9.85 (±0.82)	0.85 (± 0.03)	0.74 (±0.04)	0.12 (±0.05)	<.001
Li2^a^	24	9.38 (± 0.87)	0.85 (±0.03)	0.76 (±0.05)	0.09 (±0.05)	<.001
Li3^a^	20	9.19 (±0.84)	0.83 (±0.04)	0.71 (±0.06)	0.13 (±0.05)	<.001
Li27^a^	28	9.38 (±0.76)	0.84 (±0.03)	0.70 (±0.05)	0.16 (±0.05)	<.001
Li41^a^	19	9.24 (±0.89)	0.82 (±0.04)	0.72 (±0.05)	0.09 (±0.05)	.002
Li46^a^	29	9.72 (±1.00)	0.84 (±0.04)	0.72 (±0.05)	0.13 (±0.03)	<.001
Li53^a^	27	8.33 (±0.76)	0.83 (±0.03)	0.72 (±0.06)	0.10 (±0.08)	<.001
Li55^a^	25	9.77 (±0.84)	0.87 (±0.02)	0.78 (±0.02)	0.08 (±0.03)	.001
Li96^a^	25	9.34 (±0.83)	0.83 (±0.04)	0.71 (±0.04)	0.13 (±0.03)	<.001
LiSK^a^	18	3.57 (±0.31)^b^	0.63 (±0.03)^b^	0.67 (±0.08)	−0.08 (±0.02)	<0.001

a Population not in HWE after Bonferroni corrections.

b Outlier values based on the mean ± 1.96 × *SE*.

To collect representative samples of individuals from each wetland, dipnet sweeps were distributed equally around each wetland perimeter and interior shallow microhabitats (<0.5 m deep). When larvae were sufficiently abundant, a maximum of one individual per sweep was collected to avoid collecting full siblings. All Southern Leopard frog samples were from larval specimens, and Southern Leopard frogs were collected from all three wetlands types. Dipnet sampling was supplemented with funnel trapping and active searches for adult salamanders around wetland perimeters. Traps were distributed around the perimeter of wetlands and in shallow microhabitats and checked daily. In cases where tadpole species identification was questionable, the individuals were collected and reared in the laboratory to metamorphosis to confirm species identity. Captured individuals were euthanized in 0.5%, pH neutral‐buffered MS‐222. Tissue samples were preserved in 95% EtOH at −20°C.

### 2.3 Microsatellite marker development and analysis

DNA was extracted from Southern Leopard frog tissue using silica‐binding techniques (Lance et al., 2009) and from Dwarf salamander tissue using phenol chloroform extractions (Sambrook, Fritsch, & Maniatis, 1989). Southern Leopard frog DNA samples were screened at 11 microsatellite loci (Rasp03, Rasp07, Rasp09, Rasp10, Rasp13, Rasp17, Rasp37, Rasp45, Rasp50, Rasp53, and Rasp55) (McKee, Lance, Jones, Hagen, & Glenn, 2011b) and Dwarf salamander DNA samples at 12 microsatellite loci (Mckee, Lance, Jones, Hagen, & Glenn, 2011a) using a 3730xl Genetic Analyzer (Applied Biosystems, Foster City, CA, USA). No template controls and DNA extraction negative controls were analyzed with samples to ensure systematic contamination was not an issue. Alleles were designated with genemapper v4.0 (Applied Biosystems 2005). Approximately 10% of the samples were rerun and analyzed at each locus to estimate genotyping error rates (Table S1).

It was assumed based on the philopatric tendencies of both species (for review see Blaustein et al., 1994; Smith & Green, 2005) that the genetic compositions of breeding assemblages were similar between 2008 and 2009. Sibship among individuals was estimated for each population, in colony v2.0 (Jones & Wang, 2010). Larval samples are often biased toward particular families as they contain genetic material from successful breeders. Goldberg and Waits (2010b) found that when full siblings were collected from a population, removing all but one individual from analysis led to population and landscape genetic parameter estimates that were more similar to those calculated from adult samples. Therefore, when samples had a probability of full sibship >90%, we removed the individual with the less complete genotype (Tables S1 and S2).

Pairwise‐loci tests for linkage disequilibrium were performed with a 10,000‐step dememorization process, 10,000 batch, and 1,000‐iteration Markov chain analysis in genepop v4.2 (Raymond & Rousset, 1995; Rousset, 2008). Expected heterozygosity (*H*_e_) under Hardy–Weinberg equilibrium (HWE), observed heterozygosity (*H*_o_), the fixation index (*F*), and mean number of alleles per locus (*N*_a_) were calculated for each locus in genalex v6.503 (Table S3; Peakall & Smouse, 2006). Fixation index values can range from −1 to 1, where negative values suggest an excess of heterozygotes, while positive values suggest an excess of homozygotes, relative to expectations under HWE. Deviations from HWE for each locus were tested in genepop v4.2 using exact tests, with Bonferroni corrections. Rarefied allelic richness per locus was calculated in fstat v2.9.3.2 (Goudet, 1995).

### 2.4 Population genetic diversity and differentiation

For each population, the genetic diversity parameter rarefied allelic richness (*r*_g_) was calculated in fstat v2.9.3.2, and *H*_o_ and *F* were calculated in genalex v6.503. For both species, one of the sites (PSK) had significantly lower *r*_g_ compared to the other sites (Table 1). Subsequent analyses were performed both with and without PSK to understand how inclusion of these outlier populations affected the interpretation of associations between habitat features and genetic diversity in Dwarf salamanders and Southern Leopard frogs. Deviations from HWE were tested with exact tests using the Markov chain method in genepop v4.2 with Bonferroni corrections for multiple comparisons. Null alleles can artificially reduce *r*_g_ and *H*_o_. All loci in all populations were tested for null alleles with freeNA (Chapuis & Estoup, 2007). While mean heterozygosity often follows a normal distribution when heterozygosity values are >7.5% (Archie, 1985), we tested for statistical normality of the genetic diversity parameters with the Shapiro–Wilk normality test in r v3.2.3 (Table S4).

Genetic distances between populations were measured with *F*_ST_, calculated in genepop v4.2 (Raymond & Rousset, 1995; Rousset, 2008). Populations that demonstrate diffusive stepping‐stone model dispersal patterns should exhibit strong isolation‐by‐distance (IBD) population structure (Rousset, 1997). To test for IBD, *F*_ST_ values were linearized (*F*_ST_/(1 − *F*_ST_); Slatkin, 1993) and compared with geographic Euclidean distances between populations in genepop v4.2 using a Mantel test with 10,000 permutations.

### 2.5 Habitat and land cover characterization

Habitat and land cover variables were characterized for each sample site. arcmap 9 (ESRI 2009) was used to create circle buffers around the center of each wetland (Piha et al., 2007). Radii sizes were based on approximate spatial scales found to be significantly associated with amphibian diversity and abundance in previous studies (0.5 km, Piha et al., 2007); (1.0 km, Veysey, Mattfeldt, & Babbitt, 2011); (2.5 km, Houlahan & Findlay, 2003). National Land Cover Data (NLCD, 30‐m pixels; Homer, Huang, Yang, Wylie, & Coan, 2004) was used to calculate the percent area of each land cover feature (development, *devel*; forest, *forest*; agriculture, *ag*; and wetlands, *wtlnd*) within the buffers. Given the rural location of the study site, *devel* was a general indicator of road density. Genetic diversity parameters for both focal species were expected to be negatively associated with *devel*, which may be partial barriers to dispersal and a source of mortality (Carr & Fahrig, 2001; Gibbs, 1998; Vos & Chardon, 1998). *Forest* is necessary for upland habitat and dispersal in many other pond‐breeding amphibian species (Guerry & Hunter, 2002; Trenham & Shaffer, 2005), and therefore, genetic diversity parameters for both species were expected to be positively associated with forest cover. Genetic diversity parameters were expected to be negatively associated with *ag*, as agricultural landscapes may be partial barriers to amphibian dispersal because of the potential for water loss (Rothermel & Semlitsch, 2002). Both focal species are generally associated with aquatic habitats year‐round (Bonett & Chippindale, 2011; Cash, 2008; Means, 2008) and breed in isolated wetlands, and were therefore expected to have genetic diversity parameters positively associated with *wtlnd*. Percent area variables were arcsine square root transformed to meet assumptions of statistical distribution normality. Landscape variables at specific spatial scales are from here on referred to by a subscript (e.g., *devel*_1.0 km_ refers to development within the 1.0 km buffer).

Local habitat variables believed to be of biological relevance to both species were wetland area, isolation, and hydroperiod (*area*,* iso*, and *hydro*, respectively). Neutral genetic theory suggests that populations at larger wetlands should be larger and more genetically diverse as a result of greater carrying capacity (Antonovics, 1976). Neutral genetic theory also suggests that populations that are less isolated should be larger and more genetically diverse as a result of greater immigration rates than populations that are more isolated (Antonovics, 1976). Previous studies suggest inconsistent relationships between Dwarf salamander and Southern Leopard frog populations, and hydroperiod. While Snodgrass et al. (2000) did not find a significant relationship between hydroperiod and Dwarf salamander presence or Southern Leopard frog presence, Dwarf salamanders have been found associated with aquatic habitats year‐round (Bonett & Chippindale, 2011; Means, 2008). However, fish may be predators of Dwarf salamander larvae (Liner, 2006) Snodgrass et al. (2000), and Dwarf salamanders may therefore occur more often in wetlands with hydroperiods that are prohibitively short for establishment of fish populations. Southern Leopard frog larvae are unpalatable to local fish species and therefore may thrive in wetlands with longer hydroperiods (Babbitt et al., 2006; Baber, 2001). With the exception of PSK, a hardwood depression, all Dwarf salamanders were collected from cypress‐gum swamps, whereas Southern Leopard frogs were collected from all three wetland types. However, there was no difference in Southern Leopard frog allelic richness or heterozygosity among wetland types (Figure S1). Therefore, wetland type was not included as a predictor variable. Area was estimated from survey contours (all wetlands except PSK; Kirkman et al., 2012) and hand‐digitizing aerial photography (PSK; see Kirkman et al., 2012). Estimates were natural logarithmically transformed for subsequent analyses to meet assumptions of statistical normality. Isolation was calculated with Hanski's isolation index (Si; Hanski & Thomas, 1994) using relative distances from all 90 wetlands on Ichauway as well as 34 wetlands within a 0.25‐km buffer around Ichauway (Kirkman et al., 2012). Hydroperiod was calculated as the average number of days over a calendar year that a wetland was at least 25% full (Kirkman et al., 2012). All predictor variables were tested for statistical normality with the Shapiro–Wilk normality test in r v3.2.3 (Table S4).

### 2.6 Model selection and model averaging

We used multiple linear regression and an information theoretic approach to model genetic diversity parameters as a function of habitat features. The information theoretic approach has become more common in landscape genetic studies to investigate relationships between population genetic structure and landscape variables (Goldberg & Waits, 2010a; Nowakowski, DeWoody, Fagan, Willoughby, & Donnelly, 2015; Richardson, 2012). Pairwise population genetic diversity parameters present the issue of nonindependence of data. Therefore, genetic diversity parameters that had a single value per site were used as response variables: rarefied allelic richness (*r*_g_) and observed heterozygosity (*H*_o_). Spatial autocorrelation of predictor variables similarly violates the assumptions of statistical independence. Moran's *I* was used to examine the spatial autocorrelation of predictor variables. Multiple linear regression was performed in sam v4.0 (Rangel, Diniz Filho, & Bini, 2010).

For both *r*_g_ and *H*_o_ of each species, models were tested with all possible combinations of 1 to *N *− 1 predictor variables, where *N* is the number of populations sampled. The best supported model (i.e., top model) was selected based on the lowest Akaike's Information Criteria value corrected for small sample size (AICc; Burnham & Anderson, 2002). As many predictor variables were highly correlated (*R* ^2^ > .5), we used condition number (CN) to determine the degree to which multicollinearity was an issue within models (Lazaridis, 2007). Variable estimates are not likely affected by multicollinearity when CN is <2 therefore, models with a CN ≥ 2 were removed.

An additional analysis was performed for each genetic diversity parameter for both species to account for model selection uncertainty by averaging parameter estimates and standard errors across a confidence set of models, which were used to create a composite model that contained all predictor variables in the confidence set. The purpose of the composite model is to account for biologically relevant local or landscape variables that may not have been included in the top model. Models with differences in AICc values (ΔAICc) ≤ 2 from the top model still have substantial support and were therefore included in the confidence set (Burnham & Anderson, 2004). Limiting the models that are included in calculations of the composite model to those in the confidence set helps prevent spurious predictor variables from being included in the composite model. To account for the relative level of support for a given predictor variable to be included in the confidence set of models, relative model weights (*W*_*i*_) were calculated for the models in the confidence set containing that variable. Model‐averaged predictor variable estimates were calculated by multiplying the predictor variable estimates by the associated *W*_*i*_ *,* and summing the weighted estimates for each variable (Burnham & Anderson, 2002). Weighted unconditional standard errors were calculated in accordance with Burnham and Anderson (2004). Predictor variables in the top and composite models were considered statistically significant when the 95% confidence intervals did not cross zero.

Top models of genetic diversity for the Dwarf salamander were the same for analyses with and without PSK; however, results differed for analyses with and without PSK for the Southern Leopard frog (Table 2 and Table S5). Composite models from model averaging differed for both genetic diversity parameters for both species between analyses with and without PSK (Table 3 and Table S6). Additionally, patterns of spatial autocorrelation often differed between analyses with and without PSK, with inclusion of PSK resulting in stronger spatial autocorrelation for a number of predictor variables (Figure S2). Model selection and averaging results are therefore presented and discussed for analyses without PSK, and results from analyses with PSK are available in Tables S5 and S6.

**Table 2:** Top models of allelic richness (*r*_g_) and observed heterozygosity (*H*_o_) for the Dwarf salamander (*Eurycea quadridigitata*) and the Southern Leopard frog (*Lithobates sphenocephalus*) for populations from Ichauway, located in southwest Georgia, USA. β is the coefficient estimate. Condition number (CN) is the degree of multicollinearity in the model; when CN < 2, multicollinearity is not an issue in the model. AICc *W*_*i*_ is the model weight relative to other models with a ∆AIC ≤ 2 for the same species and genetic diversity parameter. *devel* represents development (primarily roads); *ag* represents center‐pivot agriculture and pastures; *wtlnd* represents herbaceous and wooded wetlands. These variables were calculated based on 2006 National Land Cover Data (National Land Cover Data, 30‐m pixels; Homer et al., 2004) as the percent area of each land cover feature within circular buffers with given radii

Parameter	Variable	β	95% CI	*r* ^2^	CN	AICc *W*_*i*_
Dwarf salamander						
*r*_g_	*devel*_0.5 km_	−2.70^a^, ^b^	−3.95 to −1.45	.908	1.46	0.725
	*wtlnd*_1.0 km_	9.74^a^	6.91 to 12.57			
*H*_o_	*wtlnd*_0.5 km_	0.44^a^, ^b^	0.23 to 0.65	.742	1.00	1.00
Southern Leopard frog						
*r*_g_	*ag*_2.5 km_	2.14	−0.21 to 4.49	.314	1.00	0.605
*H*_o_	*devel*_2.5 km_	−0.96	−1.97 to 0.05	.331	1.00	0.198

a 95% confidence interval of the coefficient estimate does not cross 0.

b Variable is not spatially autocorrelated.

**Table 3:** Model‐averaged estimates of local‐ and landscape‐scale predictor variables of allelic richness (*r*_g_) and observed heterozygosity (*H*_o_) in the Dwarf salamander (*Eurycea quadridigitata*) and the Southern Leopard frog (*Lithobates sphenocephalus*) for populations from Ichauway, located in southwest Georgia, USA. Estimates were calculated based on models in the confidence set (i.e*.,* all models with a ΔAICc ≤ 2)

	Variable	Model‐averaged β	Weighted unconditional *SE*	95% CI
Dwarf salamander				
*r*_g_	*devel*_0.5 km_ a b	−2.70	0.64	−3.95 to −1.45
	*wtlnd*_1.0 km_ a	9.74	1.44	6.91 to 12.57
	*wtlnd*_2.5 km_ a b	12.07	3.83	4.56 to 19.59
*H*_o_	*wtlnd*_0.5 km_ a b	0.44	0.107	0.23 to 0.65
Southern Leopard frog				
*r*_g_	*ag*_2.5 km_	2.14	1.19	−0.18 to 4.47
	*forest*_2.5 km_	−2.27	1.49	−5.18 to 0.65
*H*_o_	*iso*	0.01	0.00	0.00 to 0.01
	*devel*_0.5 km_ b	−0.14	0.08	−0.30 to 0.01
	*devel*_1.0 km_ b	−0.19	0.12	−0.42 to 0.03
	*devel*_2.5 km_	−0.96	0.52	−1.97 to 0.05
	*wtlnd*_1.0 km_	−0.15	0.11	−0.36 to 0.06
	*wtlnd*_2.5 km_	−0.34	0.19	−0.72 to 0.03

a 95% confidence interval of the coefficient estimate does not cross 0, indicating statistical significance.

b Variable is not spatially autocorrelated (*p *>* *.05), see Figure S2.

## 3 RESULTS

After removing full siblings from the analysis, the Dwarf salamander sample size ranged from 27 to 31 per wetland and the Southern Leopard frog sample size ranged from 15 to 30 (Table 1). Full siblings were removed to obtain genetic parameter estimates that were not biased toward particular families (Goldberg & Waits, 2010b). The number of alleles per locus ranged from 3 to 27 for the Dwarf salamander, and 7–29 for the Southern Leopard frog (Table S3); and among loci, the mean number of alleles across populations ranged from 2.7 to 12.9 for the Dwarf salamanders, and 4.9–16.5 for Southern Leopard frogs (Table S3). After Bonferroni corrections (Weir, 1990), there was no evidence of linkage disequilibrium for Dwarf salamander loci and the possibility of slight linkage disequilibrium between the two Southern Leopard frog loci, or inbreeding or null alleles. The high number of loci out of HWE for Dwarf salamanders (5 of 12 loci, Table S3) and Southern Leopard frogs (8 of 11 loci, Table S3) suggested null alleles or inbreeding, as indicated by the positive *F* values (Table S3). All loci had null allele frequency estimates greater than zero in at least two populations (Table S3). Null alleles can artificially reduce *r*_g_ and *H*_o_.

### 3.1 Genetic diversity parameters

All populations of both focal species differed significantly from HWE expectations after Bonferroni corrections, except three Dwarf salamander populations (Table 1). Positive values of *F* suggest an excess of homozygotes in the population relative to expectations under HWE and may indicate presence of null alleles or inbreeding. Coefficients of *F* were positive for all populations of both species, except for the PSK population of Southern Leopard frogs, and ranged from 0.050 to 0.126 for Dwarf salamanders, and −0.083 to 0.156 for Southern Leopard frogs (Table 1).

Both species had a surprising lack of variance in both genetic diversity parameters, with the exception of *r*_g_ values for PSK populations (Table 1). When PSK was removed, *r*_g_ for Dwarf salamanders ranged from 5.21 to 7.15 (*SD* = 0.58; Table 1), and 8.33–9.85 for Southern Leopard frogs (*SD* = 0.43; Table 1). Further, the variance in Southern Leopard frog *r*_g,_ when PSK was removed, was driven largely by Li53 (*r*_g_ = 8.33; Table 1), and without PSK or P53, *r*_g_ values for Southern Leopard frogs ranged from 9.19 to 9.85 (*SD* = 0.24; Table 1). Similarly, *H*_o_ values among populations of both species had low variance. Dwarf salamander *H*_o_ ranged from 0.55 to 0.64 (*SD* = 0.02), and Southern Leopard frog *H*_o_ ranged from 0.67 to 0.78 (*SD* = 0.03). Low variance in the genetic diversity parameter estimates makes it difficult to investigate associations between habitat features and genetic diversity parameter estimates as there is little variance in parameter estimates to partition.

### 3.2 Population structure and isolation by distance

Results from the analysis of genetic differentiation and isolation by distance yielded negative values of *F*_ST_ for three Southern Leopard frog pairwise comparisons, which indicates biased estimation or low statistical power. Negative *F*_ST_ values were converted to zero as they were uninterpretable from a biological perspective. As expected, Dwarf salamanders exhibited greater population structure than Southern Leopard frogs. Pairwise *F*_ST_ values with PSK ranged from 0.002 to 0.156 (mean = 0.056, *SD* = 0.037) for Dwarf salamanders, and 0–0.128 (mean = 0.031, *SD* = 0.040) for Southern Leopard frogs. When PSK was removed, the range and mean of pairwise *F*_ST_ values dropped slightly for Dwarf salamanders, ranging from 0.002 to 0.118 (mean = 0.044, *SD* = 0.030). However, removing PSK for Southern Leopard frogs resulted in a large decrease in the range and mean of pairwise *F*_ST_ values (*F*_ST_: 0.002–0.035, mean = 0.011, *SD* = 0.010).

**Figure 3:**
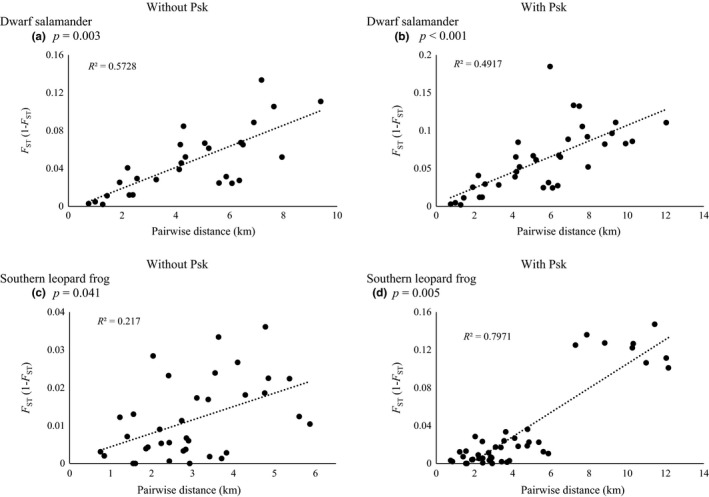
Graphs of isolation by distance for Dwarf salamander and Southern Leopard frog populations. Significant correlations between genetic distance (*F*_ST_) and geographic distance indicate populations exhibit stepping‐stone dispersal, whereby populations that are closer together are more closely related

Both species exhibited IBD, indicating that populations closer in proximity to each other were more closely related each other than those further apart. The strong correlation between genetic and geographic distance for Southern Leopard frogs was driven by PSK, as the *R* ^2^ value decreased from .797 (*p *=* *.005) to .217 (*p = *.041) after PSK was removed (Figure 3). In contrast, the strength of Dwarf salamander IBD correlations increased after PSK was removed (with PSK: *R* ^2^ = .492, *p *<* *.001; without PSK: *R* ^2^ = .573, *p *=* *.003; Figure 3), indicating this pattern was not caused by a single outlying population for Dwarf salamanders.

### 3.3 Summary of local and landscape characteristics

On average, *forest* was the most dominant and *devel* was the least dominant landscape type surrounding study wetlands for both species (Figure S3). Excluding PSK, Dwarf salamander study wetlands were on average larger than Southern Leopard frog study wetlands (mean = 4.11 ha, *SD* = 1.96 ha; mean = 1.79 ha, *SD* = 1.81 ha; respectively), more isolated (mean = −6.66, *SD* = 1.82; mean = −8.90, *SD* = 3.03; respectively), and had longer hydroperiods (mean = 193.76 days, *SD* = 21.62 days; mean = 148.93 days, *SD* = 41.22 days; respectively). When PSK was not included, most predictor variables were spatially autocorrelated at Southern Leopard frog sites, but were not spatially autocorrelated at Dwarf salamander sites (Figure S2).

### 3.4 Top models

Multicollinearity was not an issue for any of the top models of genetic diversity for either species (CN < 2 for all models). Top models of Dwarf salamander genetic diversity suggested stronger associations (greater *R* ^2^ values) with habitat variables than the top models of Southern Leopard frog genetic diversity (Table 3). Surrounding road and wetland areas were the best predictors of Dwarf salamander genetic diversity. The top model of Dwarf salamander *r*_g_ was *devel*_0.5 km_ and *wtlnd*_1.0 km_, and the top model for Dwarf salamander *H*_o_ was *wtlnd*_0.5 km_ (Table 3). Predictor variables in both Dwarf salamander top models were significant (i.e*.,* 95% confidence intervals did not cross zero), and both models were more strongly correlated with the respective diversity parameter than top models of Southern Leopard frog genetic diversity (Table 3). Top models for Southern Leopard frog genetic diversity were *ag*_2.5 km_ for *r*_g_, and *devel*_2.5 km_ for *H*_o_ (Table 3). The low *R* ^2^ values and lack of statistical significance of variables in the top models of Southern Leopard frog genetic diversity parameters (Table 3) suggested a lack or very weak relationship with the predictor variables of this study, or insufficient variance in parameter values to detect a relationship. The only predictor variables contained in any of the top models that did not exhibit significant spatial autocorrelation were *wtlnd*_0.5 km_ and *devel*_0.5 km_ for the Dwarf salamander, suggesting that the relationships between the measures of genetic diversity and other predictor variables in the associated top models may have been statistical artifacts due to spatial autocorrelation.

### 3.5 Composite models

Predictor variables in the composite models of Dwarf salamander *r*_g_ and *H*_o_ were identical to those in the respective top models, with the addition of *wtlnd*_2.5 km_ for *r*_g_ (Table 3). All associations between Dwarf salamander genetic diversity and *wtlnd* variables were positive, whereas *devel*_0.5 km_ was negatively associated with *r*_g_ (Table 3). Neither Southern Leopard frog genetic diversity parameter was significantly associated with any habitat features in the composite models (Table 3).

## 4 DISCUSSION

This study investigated the population structures of, and habitat features and spatial scales associated with genetic diversity patterns of two sympatric, pond‐breeding amphibian species that occur in the southeastern United States. Results indicated that the two species exhibited large differences in population structure and habitat features associated with genetic diversity parameters.

### 4.1 Dwarf salamander habitat associations and isolation by distance

While IBD analysis suggested that much of the population structure of Dwarf salamanders and some of the structure of Southern Leopard frog populations could be explained by distance from other wetlands, there was still unexplained variance. Based on the IBD results, we would expect wetlands that are more well‐connected to have greater genetic diversity as immigrants introduce new alleles, thereby increasing allelic richness and heterozygosity. Interestingly, our predictor variable representing isolation, Hanski's isolation index, was not significantly associated with genetic diversity of either species. However, Dwarf salamander genetic diversity was positively associated with the percent area of wetland in surrounding buffers at all three spatial scales included in our study. Given that the buffers may encompass the study wetlands themselves, as well as surrounding wetlands within the radius, these measures inherently describe a combination of wetland area and isolation. Previous studies on amphibians have suggested that wetlands within several hundred meters of each other, without significant intervening barriers to dispersal, may serve effectively as single populations (Petranka, Smith, & Floyd Scott, 2004; Veysey et al., 2011; Zamudio & Wieczorek, 2007). This may be the case for Dwarf salamander populations at Ichauway, potentially due to frequent dispersal among proximal wetlands. Similar relationships with wetland connectivity and amphibian diversity have been found in previous studies. For example, wetland presence, the number of wetlands, wetland density, and wetland area in the surrounding landscape have been found to be positively associated with amphibian species richness (Brodman et al., 2003; Houlahan & Findlay, 2003), abundance and density (Brodman et al., 2003; Hecnar & M'Closkey, 1998; Peterman, Anderson, Drake, Ousterhout, & Semlitsch, 2013), and lower levels of inbreeding (Scribner et al., 2001).

The negative association between Dwarf salamander allelic richness and percent area of roads within 0.5 km suggests that roads can have a negative effect on amphibian genetic diversity. Other studies have shown that roads are barriers to amphibian dispersal (Carr & Fahrig, 2001; Gibbs & Shriver, 2005; Reh & Seitz, 1990) and decrease abundance, species, and genetic diversity (Houlahan & Findlay, 2003; Kuhn, 1987; Reh & Seitz, 1990). The majority of roads at Ichauway are unpaved and do not experience heavy traffic, suggesting that roads can have a negative effect on amphibian genetic diversity even in a relatively rural landscape. Moreover, these results may indicate that the effects of roads were not related to mortality from vehicles traveling on roads. Rather, as suggested in previous studies, reduced moisture availability near roads may negatively impact smaller amphibian species prone to desiccation (Marsh & Beckman, 2004; Semlitsch et al., 2007). Further, drought conditions, such as those that occurred at Ichauway in 2006 through 2008 (Georgia Automated Environmental Monitoring Network 2017), may have exacerbated the effect of reduced moisture availability on or near roads (Marsh & Beckman, 2004; Semlitsch et al., 2007).

Landscape‐scale habitat features (i.e*., devel, forest, ag, wtlnd*) were more strongly associated with Dwarf salamander genetic diversity than were local features (i.e*., area, iso, hydro*). Similar results were found for spotted salamander abundance (Veysey et al., 2011). The positive relationship between Dwarf salamander allelic richness and percent wetland area within 2.5 km suggests that some degree of gene flow may occur among populations at this scale, enabling the introduction or reintroduction of alleles lost through drift. Houlahan and Findlay (2003) found a similar spatial scale to be associated with species diversity, with positive correlations between amphibian species richness and proportion of wetlands and forest cover at distances between 2.0 and 3.0 km from breeding wetlands, suggesting this spatial scale may be evolutionarily and ecologically relevant for multiple species of pond‐breeding amphibians.

### 4.2 Southern Leopard frog population structure and isolation by distance

Neither measure of genetic diversity in Southern Leopard frogs was significantly related to any of the predictor variables included in the study. This may suggest that the spatial scale of the study was not sufficient to capture metapopulation dynamics of the Southern Leopard frog. Hillman, Drewes, Hedrick, and Hancock (2014) found that dispersal distance and vagility increase with body size and that interspecific differences in vagility can contribute to differences in metapopulation structure in amphibians, which is consistent with the isolation by distances results of our study and may help explain the lack of relationship between Southern Leopard frog genetic diversity parameters and habitat features. As Ichauway is a relatively large landscape (11,800 ha), scalar results from this study are likely applicable to other large, managed landscapes. When PSK was not included, the maximum distance between Southern Leopard frog sites was 5.9 km, and results from the IBD analysis indicated very weak, albeit significant, population structure. The weak population structure of Southern Leopard frogs and the relatively homogeneous genetic diversity values of Southern Leopard frogs when PSK was not included suggests relatively high levels of gene flow among Southern Leopard populations and that at the spatial scale of our study, gene flow in Southern Leopard frogs is more important for determining genetic diversity compared to the investigated habitat features. Drought conditions exacerbated the study limitation that sample sites were selected based on being able to collect a sufficient number of our focal species during the study period, as opposed to selecting sites to encompass a range of habitat types in surrounding buffers.

### 4.3 Effect of drought

Below‐average rainfall in southwest Georgia in 2006, 2007, and 2008 (total rainfall deviated from average −10.2, −43.2, and −7.6 cm, respectively, in Baker County, Georgia (University of Georgia 2017) likely influenced how the focal species utilized the landscape prior to and during sample collection. Piha et al. (2007) found that regional‐scale variables were better predictors of Common frog (*Rana temporaria*) egg mass abundance after a period of drought, compared to the stronger relationship between landscape‐scale variables and egg mass abundance during normal weather conditions. Walls, Barichivich, Brown, Scott, and Hossack (2013) found that Mole salamander (*Ambystoma talpoideum*) occupancy rates of ponds decreased by more than 50% and local extinction rates increased in association with a 2‐year drought, potentially due to drought‐induced pond drying. Because drought rendered a number of potential study sites dry or unoccupied, the wetlands included in this study were inherently larger or had significantly longer hydroperiods than wetlands that were not included, which may have influenced the statistical relationship between genetic diversity and variables associated with wetland size and hydroperiod, as well as other habitat associations. Partially filled wetlands may have reduced the number of individuals that bred in them, thereby increasing the chance of full siblings being collected.

### 4.4 Conservation and management implications

The maintenance of population genetic diversity is widely accepted as important to the conservation and management of wildlife; however, it is an often‐overlooked component in biodiversity conservation management (Taberlet et al., 2012). This study indicates that sympatric pond‐breeding amphibian species may be differentially affected by habitat alterations. For example, targeted landscape management may be used to assist with maintaining genetically variable populations of Dwarf salamanders, and gene flow in Dwarf salamanders may be more greatly affected by habitat alterations than in Southern Leopard frogs. The low degree of population structure in the Southern Leopard frog and lack of associations between habitat features and genetic diversity parameters suggest that at the spatial scale of our study targeted landscape management may not be an necessary for maintaining or facilitating gene flow, and targeted landscape management for other species may not greatly affect rates of gene flow for Southern Leopard frogs.

Though studies have called for an integrated management of wetland complexes and intervening terrestrial habitats to conserve amphibians (Semlitsch, 2000, 2002), there is still a general tendency to manage amphibian breeding habitats as isolated units with limited buffer areas. Potentially negative impacts of even unpaved roads around wetlands on amphibian populations may also be underappreciated. Lastly, results from this study suggest that genetic diversity of sympatric amphibian species may be differentially affected by habitat types surrounding breeding wetlands, with some species more sensitive to differences in habitat types than others. Habitat management efforts focused on maintenance of genetic diversity in populations may be most effective when targeting species with stronger associations to habitat.

## CONFLICT OF INTEREST

None declared.

## DATA ACCESSIBILITY

Microsatellite data for this manuscript are archived in the online Supplementary Information.
