# Supplementary material for: Habitat predictors of genetic diversity for two sympatric wetland‐breeding amphibian species
Source: Ecol Evol. 2017 Jul 3;7(16):6271–83. doi: 10.1002/ece3.3203 (PMC5574763; doi:10.1002/ece3.3203)
Supplement: Supplementary file 2 [file ECE3-7-6271-s002.doc]

Table S1. Geographic coordinates (UTM zone 16 north) and number of individuals collected in 2008 and 2009 for dwarf salamander (Eu) and southern leopard frog (Li) populations. Numbers in parentheses indicate number of individuals that remained after removing full siblings from analysis.

| Population | UTM Coordinates | *N*_2008_ | *N*_2009_ |
| --- | --- | --- | --- |
| Eu0 | 741137E, 3466868N | 10 (10) | 21 (20) |
| Eu1 | 734139E, 3463106N | - | 31 (31) |
| Eu3 | 736048E, 3463055N | 31 (30) | - |
| Eu4 | 736666E, 3463489N | 31 (30) | - |
| Eu11 | 740856E, 3464682N | - | 31 (29) |
| Eu52 | 737998E, 3461652N | 31 (29) | - |
| Eu58 | 739473E, 3457630N | - | 30 (27) |
| Eu68 | 737331E, 3462396N | 31 (31) | - |
| EuSK | 745333E, 3458686N | 31 (28) | - |
| Li1 | 734139E, 3463106N | 1 (1) | 30 (30) |
| Li2 | 735598E, 3463773N | 2 (1) | 29 (23) |
| Li3 | 736048E, 3463055N | 18 (6) | 17 (14) |
| Li27 | 733556E, 3461669N | 16 (13) | 15 (15) |
| Li41 | 735278E, 3460956N | - | 30 (19) |
| Li46 | 736651E, 3460216N | 6 (4) | 26 (25) |
| Li53 | 738360E, 3462354N | 19 (15) | 12 (12) |
| Li55 | 738047E, 3458732N | 14 (4) | 26 (21) |
| Li96 | 734310E, 3461709N | - | 31 (25) |
| LiSK | 745333E, 3458686N | 40 (18) | - |

Table S2. Genotyping error rate estimates; calculated as the number of incorrect genotypes divided by the total number of reactions used for comparison.

| Locus | | Number of Reactions | Genotyping Error Rate |
| --- | --- | --- | --- |
| Dwarf salamander markers | | |  |
|  | Euqu01 | 63 | 0.00 |
|  | Euqu04 | 44 | 0.11 |
|  | Euqu09 | 46 | 0.00 |
|  | Euqu16 | 63 | 0.06 |
|  | Euqu17 | 64 | 0.02 |
|  | Euqu20 | 50 | 0.00 |
|  | Euqu24 | 59 | 0.00 |
|  | Euqu25 | 40 | 0.00 |
|  | Euqu31 | 58 | 0.05 |
|  | Euqu36 | 63 | 0.02 |
|  | Euqu45 | 56 | 0.11 |
|  | Euqu46 | 56 | 0.04 |
| Southern leopard frog markers | | |  |
|  | Rasp01 | 39 | 0.03 |
|  | Rasp03 | 52 | 0.00 |
|  | Rasp07 | 54 | 0.00 |
|  | Rasp09 | 28 | 0.00 |
|  | Rasp10 | 54 | 0.00 |
|  | Rasp13 | 58 | 0.00 |
|  | Rasp16 | 40 | 0.00 |
|  | Rasp17 | 60 | 0.00 |
|  | Rasp20 | 55 | 0.02 |
|  | Rasp28 | 41 | 0.10 |
|  | Rasp37 | 40 | 0.10 |
|  | Rasp42 | 52 | 0.04 |
|  | Rasp45 | 54 | 0.02 |
|  | Rasp50 | 57 | 0.02 |
|  | Rasp53 | 53 | 0.02 |
|  | Rasp55 | 50 | 0.10 |

Table S3. Characteristics of microsatellite loci used to estimate inbreeding, heterozygosity, and allelic richness in dwarf salamander (*Eurycea quadridigitata*; Euqu) and southern leopard frog (*Lithobates sphenocephalus*; Rasp) populations in southwestern Georgia (U.S.A.). Number of alleles is the total number of unique alleles across populations. *N_a_* is the mean number of alleles across populations. *H*_e_ is expected heterozygosity, calculated as Nei’s unbiased gene diversity. *H*_o_ is observed heterozygosity. *F* is the fixation index. P-value is from HWE exact tests. Null allele frequency estimates averaged across populations with suspected null alleles, number of populations contributing to the estimate in parentheses.

| Locus | | Number of alleles | *N_a_* | Allele size range (bp) | *H*_e_ | *H*_o_ | *F* | P-value | Null Allele Frequency |
| --- | --- | --- | --- | --- | --- | --- | --- | --- | --- |
| Dwarf salamander | | |  |  |  |  |  |  |  |
|  | Euqu01 | 11 | 7.556 | 279-317 | 0.797 | 0.834 | -0.069 | 0.997 | 0.002 (2) |
|  | Euqu04[[1]](#footnote-2) | 12 | 7.111 | 268-319 | 0.781 | 0.591 | 0.231 | <0.001 | 0.107 (9) |
|  | Euqu09 | 3 | 2.667 | 154-170 | 0.293 | 0.314 | -0.074 | 0.999 | 0.014 (5) |
|  | Euqu16 | 13 | 8.000 | 143-211 | 0.793 | 0.744 | 0.044 | 0.140 | 0.038 (7) |
|  | Euqu17 | 15 | 10.444 | 181-237 | 0.869 | 0.882 | -0.033 | 0.999 | 0.004 (5) |
|  | Euqu20^a^ | 4 | 2.889 | 253-269 | 0.304 | 0.182 | 0.394 | <0.001 | 0.108 (9) |
|  | Euqu24 | 10 | 6.000 | 113-150 | 0.732 | 0.665 | 0.075 | 0.016 | 0.054 (7) |
|  | Euqu25 | 4 | 3.000 | 301-324 | 0.295 | 0.302 | -0.038 | 0.999 | 0.013 (7) |
|  | Euqu31^a^ | 7 | 5.667 | 323-349 | 0.743 | 0.598 | 0.181 | 0.002 | 0.094 (8) |
|  | Euqu36^a^ | 27 | 12.889 | 297-441 | 0.890 | 0.715 | 0.179 | <0.001 | 0.096 (8) |
|  | Euqu45^a^ | 6 | 4.111 | 136-157 | 0.598 | 0.439 | 0.257 | <0.001 | 0.114 (8) |
|  | Euqu46 | 11 | 7.222 | 241-287 | 0.754 | 0.757 | -0.024 | 0.011 | 0.007 (5) |
| Southern Leopard frog | | |  |  |  |  |  |  |  |
|  | Rasp03^a^ | 18 | 11.5 | 283-339 | 0.880 | 0.785 | 0.087 | <0.001 | 0.065 (8) |
|  | Rasp07 | 8 | 4.9 | 287-308 | 0.571 | 0.616 | -0.096 | 0.245 | 0.028 (2) |
|  | Rasp09 | 18 | 11.3 | 196-372 | 0.877 | 0.899 | -0.051 | 0.849 | 0.011 (3) |
|  | Rasp10 | 17 | 11.8 | 152-224 | 0.861 | 0.794 | 0.070 | 0.014 | 0.073 (5) |
|  | Rasp13^a^ | 7 | 5.4 | 190-215 | 0.692 | 0.501 | 0.250 | <0.001 | 0.109 (10) |
|  | Rasp17^a^ | 16 | 9.8 | 230-290 | 0.861 | 0.804 | 0.044 | <0.001 | 0.048 (7) |
|  | Rasp37^a^ | 29 | 16.5 | 212-303 | 0.908 | 0.788 | 0.100 | <0.001 | 0.067 (9) |
|  | Rasp45^a^ | 19 | 12.7 | 159-228 | 0.884 | 0.677 | 0.209 | <0.001 | 0.118 (9) |
|  | Rasp50^a^ | 18 | 9.6 | 421-493 | 0.819 | 0.635 | 0.230 | <0.001 | 0.102 (10) |
|  | Rasp53^a^ | 19 | 11.8 | 266-335 | 0.887 | 0.791 | 0.082 | <0.001 | 0.055 (9) |
|  | Rasp55^a^ | 23 | 10.8 | 152-242 | 0.787 | 0.673 | 0.098 | <0.001 | 0.090 (9) |

Table S4. Results from Shapiro-Wilk normality tests for predictor and response variables.

|  |  | | Without Psk | | With Psk | |
| --- | --- | --- | --- | --- | --- | --- |
|  | Species/Parameter | | w | p-value | w | p-value |
| Dwarf salamander | | |  |  |  |  |
|  | *r_g_* | | 0.865 | 0.136 | 0.843 | 0.063 |
|  | *H_o_* | | 0.897 | 0.272 | 0.892 | 0.207 |
|  | *F*_IS_ | | 0.952 | 0.735 | 0.963 | 0.826 |
|  | *area* | | 0.973 | 0.920 | 0.867 | 0.115 |
|  | *hydro* | | 0.878 | 0.180 | 0.739 | 0.004 |
|  | *iso* | | 0.961 | 0.816 | 0.942 | 0.604 |
|  | *ag* | |  |  |  |  |
|  |  | 0.5km | 0.952 | 0.730 | 0.941 | 0.596 |
|  |  | 1.0km | 0.936 | 0.576 | 0.956 | 0.751 |
|  |  | 2.5km | 0.857 | 0.112 | 0.861 | 0.099 |
|  | *devel* | |  |  |  |  |
|  |  | 0.5km | 0.898 | 0.275 | 0.888 | 0.190 |
|  |  | 1.0km | 0.936 | 0.576 | 0.929 | 0.472 |
|  |  | 2.5km | 0.816 | 0.043 | 0.799 | 0.020 |
|  | *forest* | |  |  |  |  |
|  |  | 0.5km | 0.962 | 0.826 | 0.917 | 0.371 |
|  |  | 1.0km | 0.919 | 0.426 | 0.924 | 0.427 |
|  |  | 2.5km | 0.914 | 0.381 | 0.914 | 0.342 |
|  | *wtlnd* | |  |  |  |  |
|  |  | 0.5km | 0.899 | 0.284 | 0.887 | 0.187 |
|  |  | 1.0km | 0.939 | 0.597 | 0.933 | 0.512 |
|  |  | 2.5km | 0.896 | 0.268 | 0.809 | 0.026 |
|  |  | |  |  |  |  |
| Southern Leopard frog | | |  |  |  |  |
|  | *r_g_* | | 0.851 | 0.076 | 0.558 | 0.000 |
|  | *H_o_* | | 0.903 | 0.272 | 0.964 | 0.825 |
|  | *F*_IS_ | | 0.926 | 0.441 | 0.719 | 0.002 |
|  | *area* | | 0.929 | 0.473 | 0.960 | 0.785 |
|  | *hydro* | | 0.938 | 0.559 | 0.914 | 0.309 |
|  | *iso* | | 0.922 | 0.411 | 0.955 | 0.725 |
|  | *ag* | |  |  |  |  |
|  |  | 0.5km | 0.917 | 0.368 | 0.964 | 0.825 |
|  |  | 1.0km | 0.927 | 0.450 | 0.984 | 0.983 |
|  |  | 2.5km | 0.962 | 0.820 | 0.946 | 0.617 |
|  | *devel* | |  |  |  |  |
|  |  | 0.5km | 0.858 | 0.091 | 0.837 | 0.041 |
|  |  | 1.0km | 0.840 | 0.058 | 0.857 | 0.071 |
|  |  | 2.5km | 0.813 | 0.029 | 0.848 | 0.056 |
|  | *forest* | |  |  |  |  |
|  |  | 0.5km | 0.899 | 0.247 | 0.950 | 0.664 |
|  |  | 1.0km | 0.932 | 0.496 | 0.972 | 0.905 |
|  |  | 2.5km | 0.949 | 0.684 | 0.960 | 0.782 |
|  | *wtlnd* | |  |  |  |  |
|  |  | 0.5km | 0.976 | 0.941 | 0.967 | 0.859 |
|  |  | 1.0km | 0.915 | 0.354 | 0.892 | 0.177 |
|  |  | 2.5km | 0.801 | 0.021 | 0.745 | 0.003 |

Table S5. Top models of allelic richness (*r*_g_) and observed heterozygosity (*H*_o_) for the dwarf salamander (*Eurycea quadridigitata*) and the southern leopard frog (*Lithobates sphenocephalus*; N=10) when the outlier site, Psk, was included. β is the coefficient estimate. Condition number (CN) is the degree of multicollinearity in the model; when CN < 2, multicollinearity is not an issue in the model. AICc *W*_i_ is the model weight relative to other models with a ∆AIC ≤ 2 for the same species and genetic diversity parameter. *devel* represents development (primarily roads); *ag* represents center pivot agriculture and pastures; *wtlnd* represents herbaceous and wooded wetlands. These variables were calculated based on 2006 National Land cover Data (NLCD, 30-m pixels; Homer et al. 2004) as the percent area of each land cover feature within circular buffers with given radii. *iso* represents isolation; calculated with Hanski’s isolation index (S_i_; Hanski and Thomas 1994).

| Parameter | |  | Variable | β | SE | 95% CI | r^2^ | CN | AICc *W*_i_ |
| --- | --- | --- | --- | --- | --- | --- | --- | --- | --- |
| Dwarf Salamander | | | |  |  |  |  |  |  |
|  | *r*_g_ |  | *devel*_0.5km_ | -2.60[[2]](#footnote-3)[[3]](#footnote-4) | 0.54 | -3.65 - -1.54 | 0.96 | 1.06 | 1.00 |
|  |  |  | *wtlnd*_1.0km_ | 9.29^a^ | 0.81 | 7.69 – 10.88 |  |  |  |
|  | *H_o_* |  | *wtlnd*_0.5km_ | 0.14 | 0.10 | -0.05 - 0.32 | 0.22 | 1.00 | 0.21 |
| Southern Leopard Frog | | | |  |  |  |  |  |  |
|  | *r*_g_ |  | *devel*_2.5km_ | -61.36^a^ | 24.57 | -109.51 - -13.20 | 0.80 | 1.76 | 0.55 |
|  |  |  | *wtlnd*_2.5km_ | 25.01^a^ | 4.68 | 15.85 - 34.18 |  |  |  |
|  | *H_o_* |  | *devel*_2.5km_ | -30.76 | 45.69 | -120.31 – 58.80 | 0.69 | 1.64 | 1.00 |
|  |  |  | *forest*_0.5km_ | 5.30^b^ | 3.18 | -0.93 – 11.52 |  |  |  |

Table S6. Model averaged estimates of landscape scale predictor variables of allelic richness (*r_g_*) and observed heterozygosity (*H_o_*) in the dwarf salamander (*Eurycea quadridigitata*) and the southern leopard frog (*Lithobates sphenocephalus*) with the outlier site, Psk, included. Estimates were calculated based on models in the confidence set (i.e. all models with a ΔAICc ≤ 2).

|  | Variable | Model averaged β | Weighted Unconditional SE | 95% CI |
| --- | --- | --- | --- | --- |
| Dwarf Salamander | | |  |  |
| *r*_g_ | *devel*_0.5km_[[4]](#footnote-5)[[5]](#footnote-6) | -2.60 | 0.54 | -3.65 - -1.54 |
|  | *wtlnd*_1.0km_^a^ | 9.29 | 0.81 | 7.69 - 10.88 |
|  |  |  |  |  |
| *H_o_* | *ag*_0.5km_ | -0.03 | 0.04 | -0.10 – 0.05 |
|  | *ag*_0.5km_ | -0.03 | 0.04 | -0.11 – 0.05 |
|  | *devel*_0.5km_^b^ | -0.04 | 0.07 | -0.17 – 0.09 |
|  | *forest*_0.5km_^b^ | 0.03 | 0.05 | -0.08 – 0.13 |
|  | *forest*_1.0km_ | 0.05 | 0.06 | -0.06 – 0.16 |
|  | *forest*_2.5km_^b^ | 0.06 | 0.09 | -0.12 – 0.24 |
|  | *wtlnd*_0.5km_ | 0.14 | 0.10 | -0.05 – 0.32 |
|  | *wtlnd*_1.0km_ | 0.10 | 0.10 | -0.08 – 0.29 |
|  | *wtlnd*_2.5km_ | 0.09 | 0.11 | -0.13 – 0.32 |
|  |  |  |  |  |
| Southern Leopard Frog | |  |  |  |
| *r*_g_ | *devel*_2.5km_^a^ | -61.36 | 24.57 | -109.51 – 13.20 |
|  | *wtlnd*_2.5km_ | 22.38 | 12.02 | -1.19 – 45.94 |
|  |  |  |  |  |
| *H_o_* | *devel*_2.5km_ | -30.76 | 45.69 | -120.31 – 58.80 |
|  | *forest*_0.5km_^b^ | 5.30 | 3.18 | -0.93 – 11.52 |

Figure S1. Southern Leopard frog (*Lithobates sphenocephalus*) rarefied allelic richness (a) and heterozygosity (b) by wetland type, excluding Psk. N=3 for all wetland types, error bars are 95% CI (1.96*SD).

a)


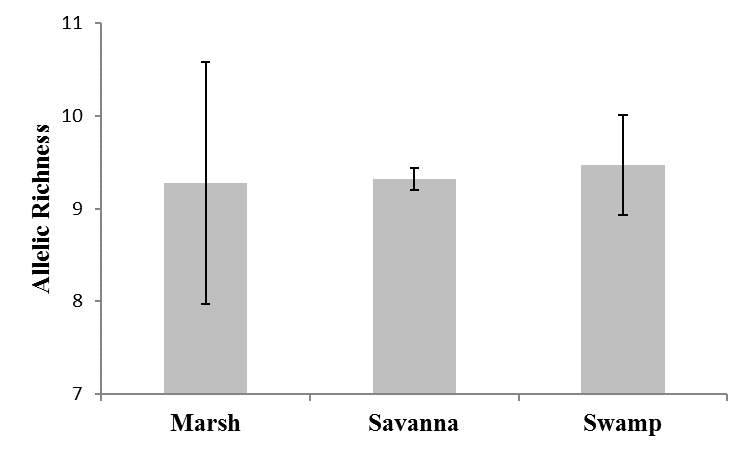


b)


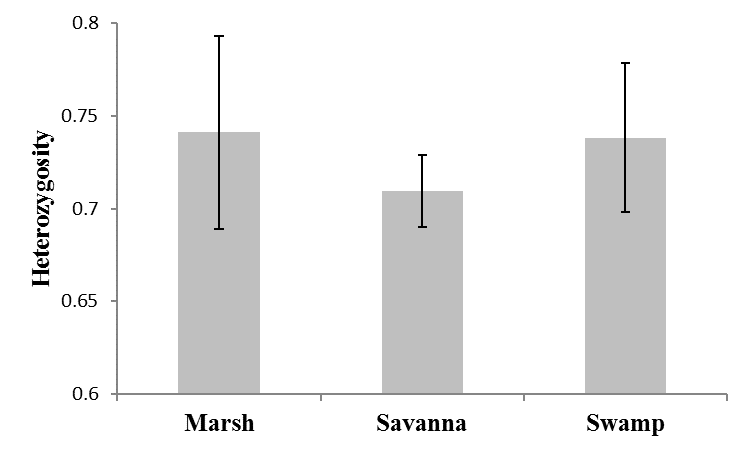


Figure S2. Moran’s I correlograms of Southern Leopard frog (a – q) and Dwarf salamander (r – ah) predictor and response variables, both with Psk (∆) and without Psk (□). Moran’s I values that significantly deviate from 0 (evidence of spatial autocorrelation) when alpha = 0.05 are filled in (▲ when Psk was included, or ■ when Psk was excluded).

|  | a) *ag* 0.5km | b) *ag* 1.0km | c) *ag* 2.5km |
| --- | --- | --- | --- |
| Moran’s I | 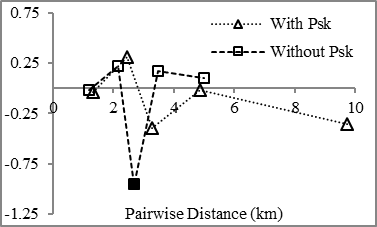 | 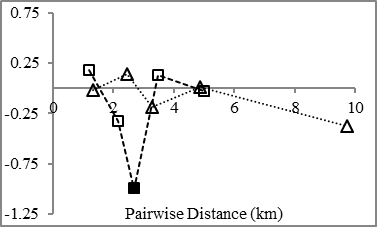 | 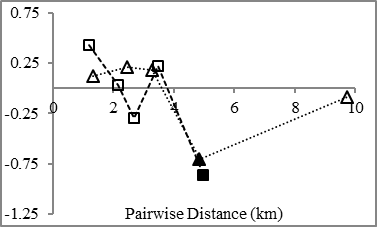 |
|  | d) *devel* 0.5km | e) *devel* 1.0km | f) *devel* 2.5km |
| Moran’s I | 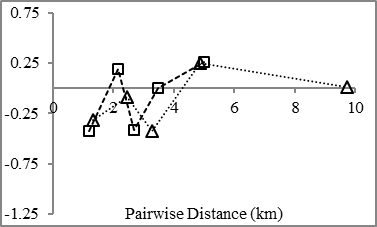 | 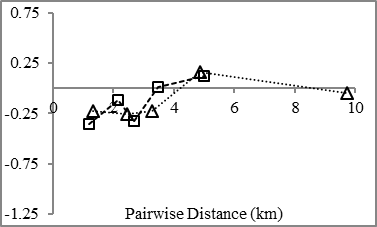 | 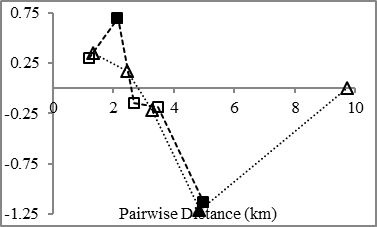 |
|  | g) *forest* 0.5km | h) *forest* 1.0km | i) *forest* 2.5km |
| Moran’s I | 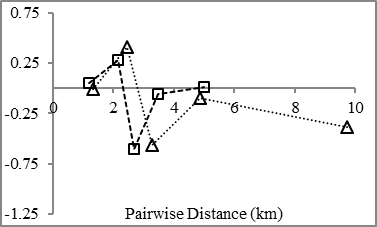 | 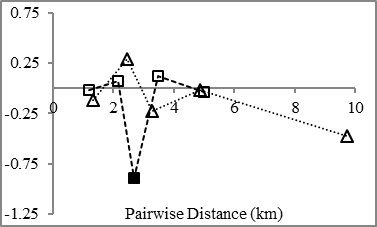 | 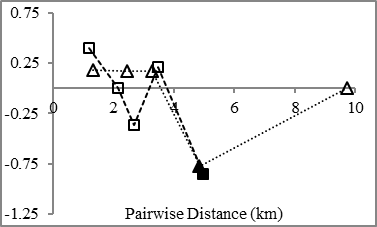 |
|  | j) *wtlnd* 0.5km | k) *wtlnd* 1.0km | l) *wtlnd* 2.5km |
| Moran’s I | 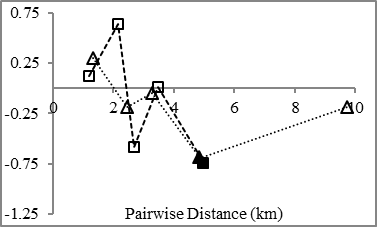 | 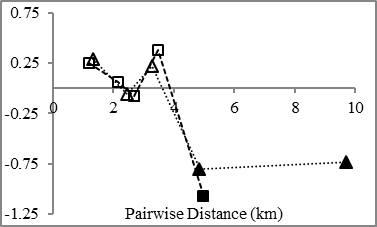 | 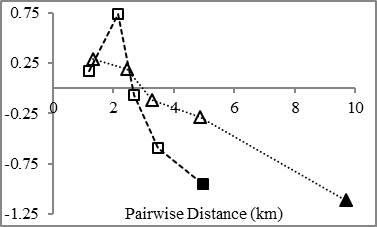 |
|  | m) *area* | n) *hydro* | o) *iso* |
| Moran’s I | 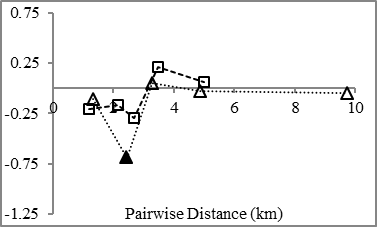 | 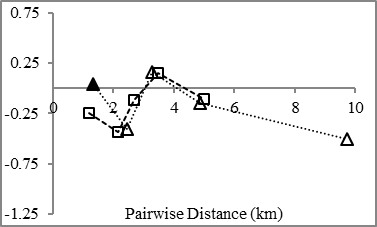 | 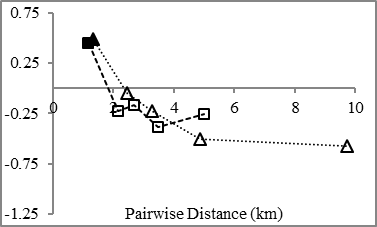 |
|  | p) *r_g_* | q) *H_o_* |  |
| Moran’s I | 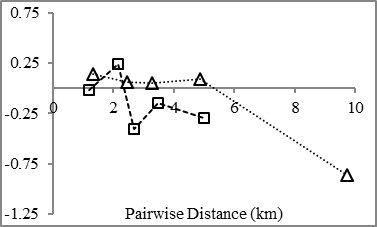 | 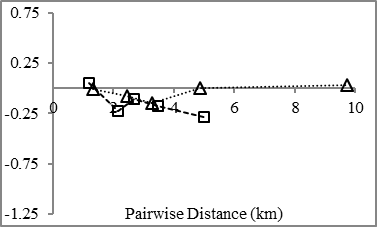 |  |
|  | r) *ag* 0.5km | s) *ag* 1.0km | t) *ag* 2.5km |
| Moran’s I | 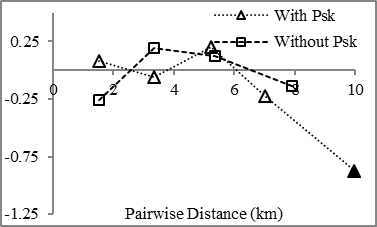 | 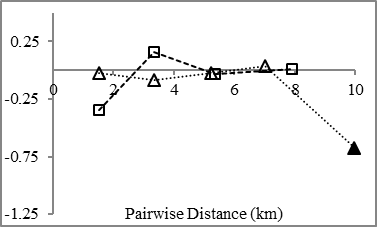 | 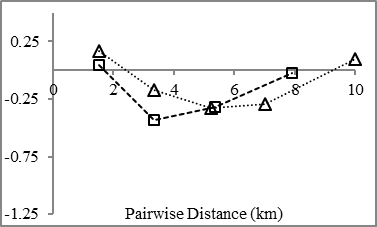 |
|  | u) *devel* 0.5km | v) *devel* 1.0km | w) *devel* 2.5km |
| Moran’s I | 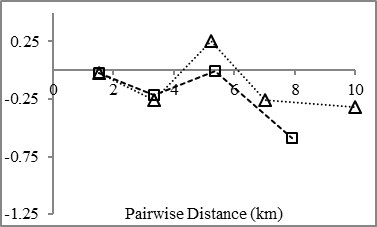 | 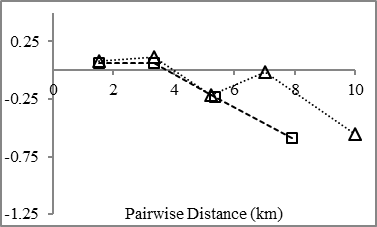 | 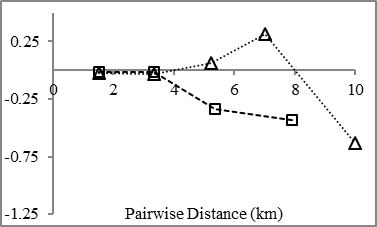 |
|  | x) *forest* 0.5km | y) *forest* 1.0km | z) *forest* 2.5km |
| Moran’s I | 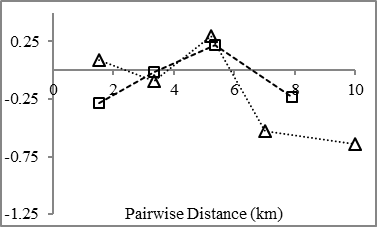 | 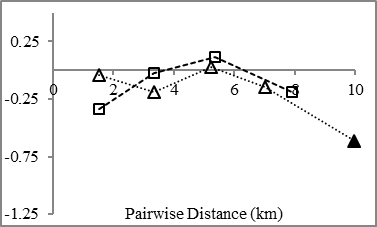 | 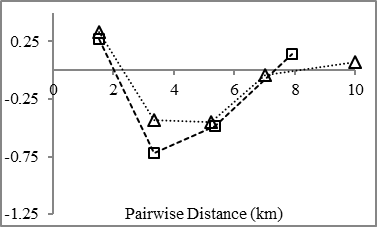 |
|  | aa) *wtlnd* 0.5km | ab) *wtlnd* 1.0km | ac) *wtlnd* 2.5km |
| Moran’s I | 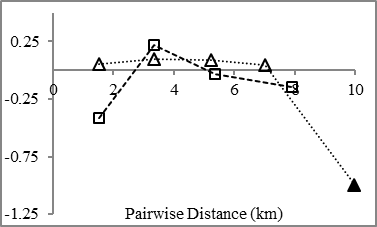 | 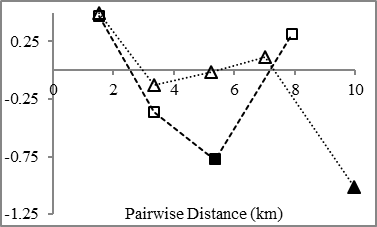 | 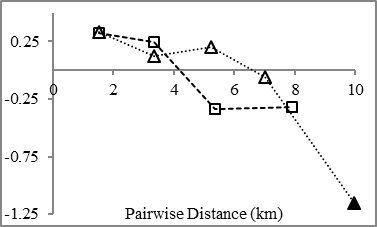 |
|  | ad) *area* | ae) *hydro* | af) *iso* |
| Moran’s I | 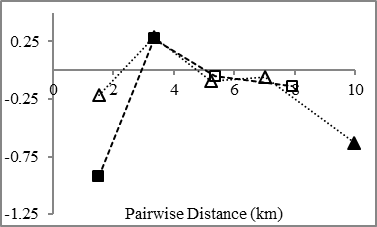 | 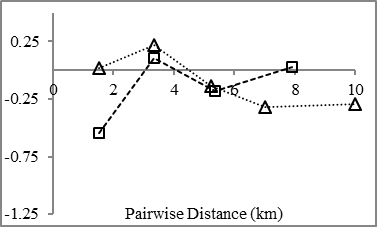 | 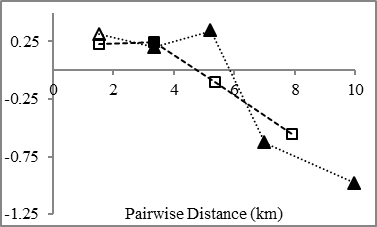 |
|  | ag) *r_g_* | ah) *H_o_* |  |
| Moran’s I | 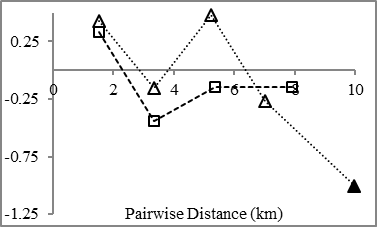 | 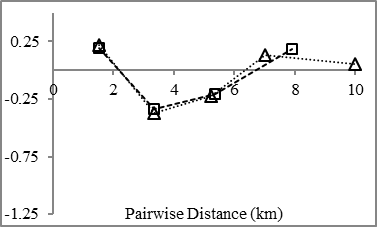 |  |

Figure S3. Percent area of landscape-scale predictor variables within buffers across sites. Blue bars are for 0.5km buffers, red bars are for 1.0km buffers, and green bars are for 2.5km buffers.

| a) Dwarf salamander, agriculture | b) Dwarf salamander, development |
| --- | --- |
| 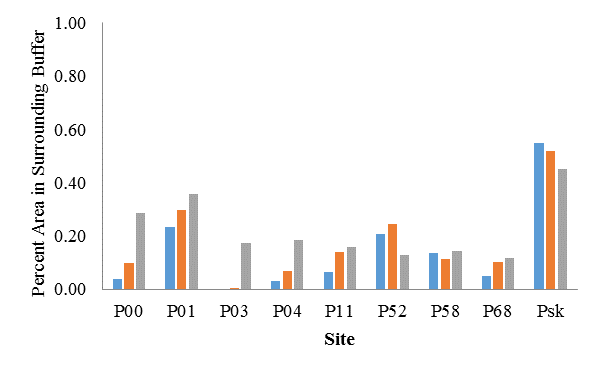 | 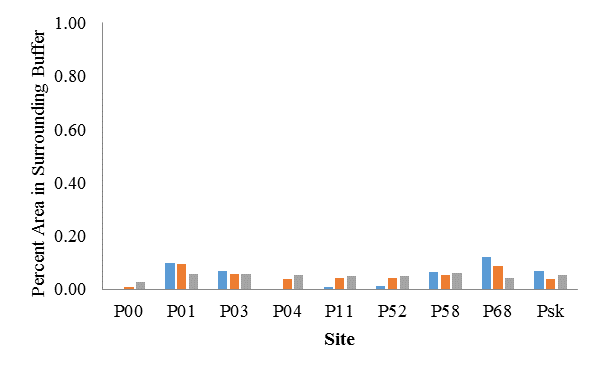 |
| c) Dwarf salamander, forest | d) Dwarf salamander, wetland |
| 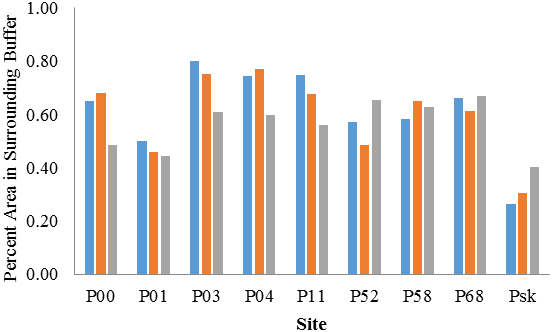 | 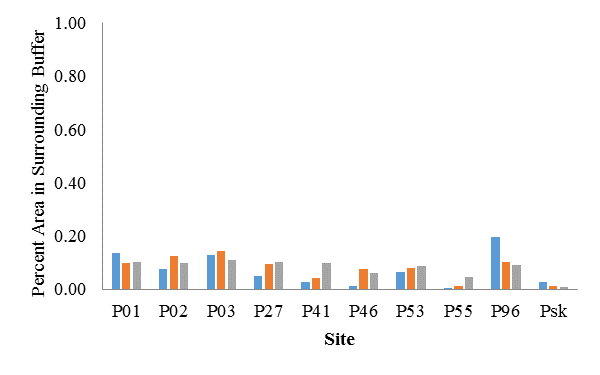 |
| e) Southern leopard frog, agriculture | f) Southern leopard frog, development |
| 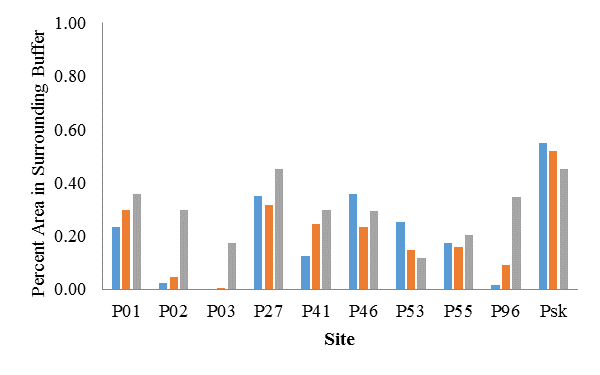 | 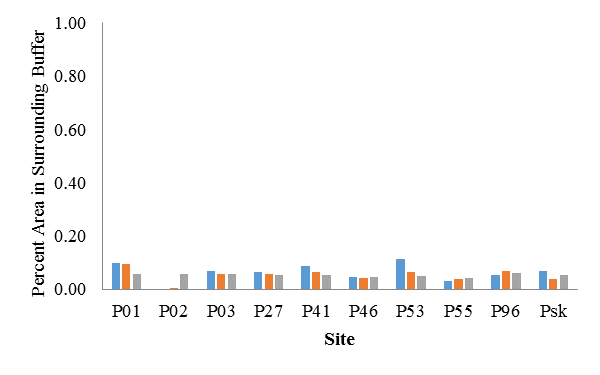 |
| g) Southern leopard frog, forest | h) Southern leopard frog, wetland |
| 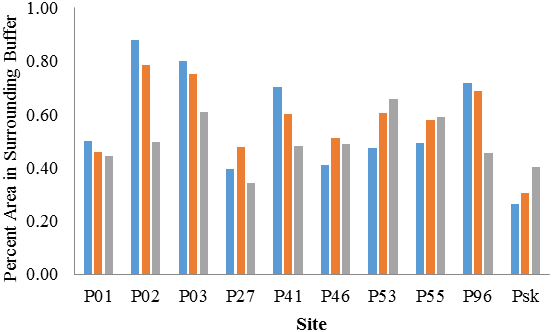 | 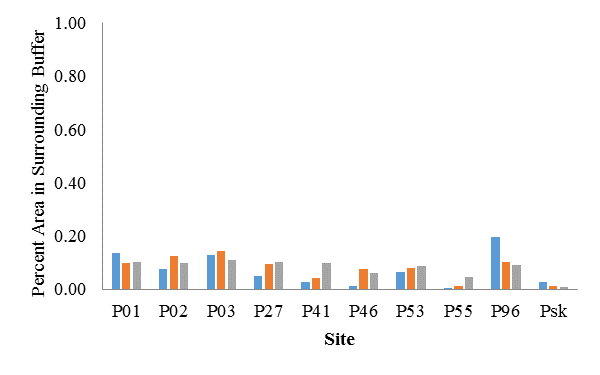 |

1. Out of HWE after Bonferroni correction for multiple comparisons, *P*  < 0.004 for dwarf salamanders, *P* < 0.006 for southern leopard frogs [↑](#footnote-ref-2)
2. 95% confidence interval of the coefficient estimate does not cross 0, indicating statistical significance. [↑](#footnote-ref-3)
3. Variable is not spatially autocorrelated (*P* > 0.05), see Fig S2. [↑](#footnote-ref-4)
4. 95% confidence interval of the coefficient estimate does not cross 0, indicating statistical significance. [↑](#footnote-ref-5)
5. Variable is not spatially autocorrelated (*p* > 0.05), see Fig. S2 [↑](#footnote-ref-6)
